# Pathways to care: a case study of traffic injury in Vietnam

**DOI:** 10.1186/s12889-021-10539-9

**Published:** 2021-03-16

**Authors:** Thanh Tam Tran, Adrian Sleigh, Cathy Banwell

**Affiliations:** 1 National Centre for Epidemiology and Population Health, College of Health and Medicine, The Australian National University, Building 62 Mills Road, Canberra, ACT 2601 Australia; 2 Canberra Hospital, Canberra, ACT Australia

**Keywords:** Emergency medical services, EMS, Prehospital care, Traffic injury, Vietnam, Lower-middle income country, Three-delay model of care

## Abstract

**Background:**

Traffic injuries place a significant burden on mortality, morbidity and health services worldwide. Qualitative factors are important determinants of health but they are often ignored in the study of injury and corresponding development of prehospital Emergency Medical Services (EMS), especially in developing country settings. Here we report our research on sociocultural factors shaping pathways to hospital care for those injured on the roads and streets of Vietnam.

**Methods:**

Qualitative fieldwork on pathways to emergency care of traffic injury was carried out from March to August 2016 in four hospitals in Vietnam, two in Ho Chi Minh City and two in Hanoi. Forty-eight traffic injured patients and their families were interviewed at length using a semi-structured topic guide regarding their journey to the hospital, help received, personal beliefs and other matters that they thought important. Transcribed interviews were analysed thematically guided by the *three-delay model* of emergency care.

**Results:**

*Seeking care* was the first delay and reflected concerns over money and possessions. The family was central for transporting and caring for the patient but their late arrival prolonged time spent at the scene. *Reaching care* was the second delay and detours to inappropriate primary care services had postponed the eventual trip to the hospital. Ambulance services were misunderstood and believed to be suboptimal, making taxis the preferred form of transport. *Receiving care* at the hospital was the third delay and both patients and families distrusted service quality. Request to transfer to other hospitals often created more conflict. Overall, sociocultural beliefs of groups of people were very influential.

**Conclusions:**

Analysis using the *three-delay model* for road traffic injury in Vietnam has revealed important barriers to emergency care. Hospital care needs to improve to enhance patient experiences and trust. Socioculture affects each of the three delays and needs to inform thinking of future developments of the EMS system, especially for countries with limited resources.

**Supplementary Information:**

The online version contains supplementary material available at 10.1186/s12889-021-10539-9.

## Background

Transition from war to peace involves many societal transformations including the conversion of emergency health services from a military to a civilian format. Following World War II, Western countries developed emergency health services (EHS) using two principal models, generally characterised today as a doctor-centred ‘stay and play’ (Franco-German) or a paramedic ‘scoop and run’ (Anglo-American) [1]. By now every high income country (HIC) has fine-tuned its own system and has succeeded in stabilising and transporting most people needing emergency care to health services in remarkably short time. The Franco-German and Anglo-American models continue to generate disagreements over the ideal system but it seems in general that both work rather well [2]. In some settings, a hybrid system of play and transport had developed initially supported by radiotelemetry of ECG. Now resuscitative support during the transfer with helicopter ambulances and mobile surgeons are now widespread.

Unfortunately, such post war EHS development was initially not possible in most resource constrained lower middle-income countries (LMICs). These countries cannot simply adopt a model based on medical stabilisation or rapid transport as neither is possible yet [3]. Ambulances are not equipped to support stabilisation and roads are gridlocked by traffic that prevents rapid transport [4]. Even if such resources were available, their populations remain constrained by complex traditional sociocultural behaviour when responding to a need for emergency care. Issues such as distrust of the system and misunderstanding of the service intention underscored patient’s negative experiences [5]. Cultural beliefs about causes of sickness and remedial measures shaped service-seeking behaviours. Consequently, LMICs have mostly failed to convert their military experience – very considerable for Vietnam – into a trusted emergency health care.

Indeed in Vietnam today the civilian EHS is still not functioning well. More than 90% of road injured victims do not receive first aid on-site [6]. Less than 10 out of 63 provinces/cities have an EMS system, and the response when called is very limited due to shortage of qualified staff and resources [6]. Growing congestion on Vietnam’s roads poses another challenge to the activities of emergency service, especially in big cities such as Ho Chi Minh City and Ha Noi where traffic congestion is a major hindrance to a swift response to incidents. Less than 10% of patients are transported by ambulance. Most patients are carried to hospital on motorbikes or in some cases by cars or taxi [6]. Under these conditions, 29% of Vietnamese road traffic deaths occurred on-site, 42% of all traffic deaths happened before the arrival at hospital, and 80% of all traffic deaths took place on the same day of injury [7].

Our study responds to challenges faced by Vietnam as it attempts to develop an accessible, affordable and acceptable EHS. Our exploration examines sociocultural factors that contribute to non-use of EHS and their relationship to the three-delay model of emergency care – first *seeking* care, second *reaching* care*,* and third *receiving* care [8, 9]. The three delay model assumes a lack of timely and adequate care as the foundation of emergency death and shifts the blame away from the patient [8, 9]. This staging fits well with corresponding death and injury data from Vietnam and casts light on the factors shaping patient pathways to care following traffic incidents. For all three delays, socioeconomic and cultural factors interact with health system features to determine accessibility and quality of the health care. Sociocultural attributes, such as perceptions, beliefs, practices, social situations, and political contexts all affect injury outcome and distribution yet cannot be readily captured with quantitative methods alone [10]. Until recently, most injury research has had a clinical focus on counting cases, measuring needs and evaluating interventions [10]. However, there are limits to what can be measured and counted.

The overarching aim of our study is to understand the complex interplay between traffic injuries, societal behaviours, cultural values and prehospital emergency care in Vietnam. The objective is to identify sociocultural forces that influence post-crash management strategies. We will then learn how to enhance health system responsiveness to patient’s needs and outcomes to the growing problem of traffic injury.

## Methods

This study adopted a focused ethnographic approach using in-depth interviews and direct observation [11, 12]. This type of ethnographic method is suited to the nature of the research aim and is flexible enough to allow for the exploration of rich cultural contexts yet is practical given the time constraint [12]. The fieldwork took place from March to August 2016 in Ha Noi and Ho Chi Minh City (HCMC), Vietnam’s two biggest cities, one in the north and one in the south. In each city, two hospitals were chosen in consultation with senior staff at the city’s Department of Health on the basis of availability of logistic support. In Ho Chi Minh City, both hospitals were located in the same district; one was a tertiary hospital (level I) and the other a district hospital (level II). In Ha Noi, both hospitals were tertiary, one in the city centre and the other on the outskirt.

The lead author is a medical graduate, PhD scholar and a native speaker of Vietnamese (TT). She spent approximately 2 weeks in orthopaedic and neurosurgical wards in each of the four study hospitals, recruiting and interviewing 10 patients and their family members, if they were present. In total, 48 interviews (40 patients and additional families) were conducted (Table 1). We noticed that there was an imbalance between males and females at some study locations. This was partially due to the hospital characteristics of being located in heavy industrial area, thus more male patients. As well, some hospitals segregated their patients based on gender; if the first few patients agreed or disagreed to participate, all other patients shared the same room might do the same.

**Table 1:** Characteristics of participants in traffic injury study, Vietnam

		Hospital 1 (level 2 – Ho Chi Minh City)	Hospital 2 (level 1 – Ho Chi Minh City)	Hospital 3 (level 1 – Central Ha Noi)	Hospital 4 (level 1 – Ha Noi outskirt)
Gender	Male	9	2	9	6
	Females	1	7	2	4
Age groups	20–40	4	5	7	5
	40–60	4	1	3	3
	60+	2	3	1	2
Mode of transport at time of injury	Motorbike (rider)	6	7	10	10
	Motorbike (passenger)	3	0	0	0
	Pedestrian	1	2	1	0
Injury profile^a^	Head injury	5	0	3	3
	Upper limb	4	4	2	1
	Lower limb	2	6	8	6
	Others ^b^	0	1	1	0
No. Interviews with family member/friend		4	3	1	0
Patient ward		Orthopaedic Neurosurgery	Orthopaedic	Orthopaedic	Orthopaedic Neurosurgery
Transport to first hospital/clinic		7 motorbikes 1 ambulance 2 private car/taxi	6 motorbikes 3 private car/taxi	2 motorbike 8 private car/taxi 1 ambulance	5 motorbike 5 private car/taxi

^a^Total number of injuries might not add up to number of participants as some suffered from multiple injuries

^b^incl. Spine and pelvic injury/fracture

Patients were recruited if they were injured on the road as a result of vehicle collisions, vehicle malfunctioning, or rough terrain. All patients sampled were inpatient at one of the four research hospitals and overall more than 80% of those approached agree to participate. The eligible patients were introduced to TT by a nurse or a doctor. All those studied consented to participate in the study. The in-depth interviews were semi-structured using an open-ended list of topics that allowed for the inclusion of a wide range of subjects. Information elicited pertained to how the crash happened, the patient’s pathway to care and treatments received at the hospital. Interviews typically lasted 1 h and were summarized by extensive note-taking. They were also digitally recorded and subsequently fully transcribed in Vietnamese by TT.

Thematic analysis of the transcripts then followed in five steps [13]. First, the lead author read and reread transcripts to familiarise herself with the data. Second, guided by the literature on emergency care and three delay model, she developed coding categories with respect to perceptions, attitudes and knowledge of the participants and their description of events. Third, using ATLAS ti 8, TT coded each transcript and gradually developed themes relevant to the study. Fourth, the themes within and across patient accounts were compared with the literature to identify emerging concepts that shed light onto patient pathways to care.

Finally, the themes were explored to ascertain how they aligned with the three delay framework developed by Calvello and colleagues [8] to understand interruptions in seeking, reaching and receiving care. The analysis is qualitative and based on experiences of 40 patients and their perceptions of care. Therefore, this paper is based on a relatively large sample for sociocultural health research, and increases understanding of a patient’s view of the entire process from injury to inpatient care. The paper does not include assessment of the appropriateness of care as this would be beyond the scope of the research. We use pseudonyms in our report to protect the participants’ identity.

Vietnamese terminology of “traffic accident” has been adopted for this study and is equivalent to the internationally more frequent “traffic crash” or “traffic incident”.

## Results

We incorporated the three delay model into our analysis as follows. For *seeking care* we gathered data beginning with the scene of the crash including the process whereby the patient or their care-givers realised that hospital treatments were required. Our *reaching care* data described the events experienced by patients until they reached the first hospital. *Receiving care* data included problems arising after the patients had been admitted to a hospital and transferral between hospitals. For each of the three delays, we described socio-cultural and system factors that contributed to delays. Major themes identified were summarised in Table 2 .

**Table 2:** Major themes identified from the findings listed based on the three delay model

Seeking care	Reaching care	Receiving care
Trust in hospital treatment Previous experience Perceived injury severity Financial concern	EMS/ambulance role Taxi/private transport Other care services	Admission process Transfer among facilities Misdiagnosis Trust in hospital Cost and insurance

### Seeking care

Many patients delayed seeking care because they were wary of hospitals and their treatment regimes. Their fears were based on previous experiences or stories that included an inter-play of personal, social and financial concerns. Indeed, sociocultural issues were identified by almost all interviewees and included ideas, beliefs and reinforced by their families. As was the case with *Kim.* She fractured her patella when she tripped on a bumpy road but refused to go to the hospital until her brother, a doctor forced her to go. He explained that:

First, she is frightened of surgery and of dying; then she is scared that she had to share a room with other people and worried that the service would not be good. So I have to assure her that she would get a private room for herself [...] I also called my contacts here and they operated on her immediately as we got to the hospital. (Case 24, Kim’s brother).

Another interviewee reported having a comminuted fracture forcing him to go to the hospital.

I have a comminuted fracture so I have to go to the hospital. If it was a simple fracture I would have gone to the traditional healers instead. They [traditional healers] use herbs so it does not affect my health. This hospital uses antibiotics which make my leg shrink. The antibiotics are really bad for your health. (Case 36, Male, 30s, calcaneus fracture).

It is noteworthy that patients were generally sceptical of hospital treatments and some of the believe was based on sociocultural experiences. Illustrating financial anxieties, many patients used the popular proverb – *“Tiền mất, tật mang”* [literally: losing money, sustaining disability] to describe going to hospital as a wasted effort with more monetary loss than health gain.

Building on other concerns, the trip to hospital was often postponed because patients misjudged the severity of their injury, preferring to self-treat because it was cheaper and more convenient. One patient, *Hong* (M, 50s, clavicle dislocation) reported that he only went to hospital the day after traffic crash when the pain became unbearable and medicinal oil that he had applied did not seem to have much effects.

I did not think the injury was so serious. I thought it was just swollen from all the bruises. I thought to myself to do an X-ray just in case. It turned out that the clavicular joint was dislocated and the doctor said I need surgery to fix that. (Hong, Male, 50s, clavicular dislocation).

Another major reason for delay was that the patients preferred to wait for friends or family to arrive at the scene to take charge of their belongings. Motorbikes are the most common form of private transport with riders highly prone to traffic related injuries. For many Vietnamese people, a motorbike is not only their most valuable asset but also a means of making a living. Thus, patients were reluctant to leave their motorbike at the crash scene for fear of it being stolen. Leaving a vehicle with a stranger required more trust than most people had. For example, *Chinh* explained that he would rather wait 30 min for his friend to arrive rather than accepting the assistance of a bystander.

I am very wary of swindlers. They are very cruel; they have no morals. I am worried that my bike would be stolen. So the moment I fell on the ground, I immediately tried to pick up the bike and took out the keys. (Chinh, 20s, male, construction worker, torn ligaments).

The same sentiments were echoed by others, irrespective of socio-economic status or location, although it reflects the general poor economic status of the country.

### Reaching care

Experiences and decisions about reaching care also exemplified a complex mix of sociocultural and economic considerations, including the difficulties of travel in Vietnam and cost. In contrast to economically developed Western countries, ambulances are rarely used in Vietnam with only two patients out of 40 in our sample transported to the hospital in one. Ambulances were distrusted or only used for life threatening conditions.

You only call [the ambulance] if you are very sick; if you cannot breathe and need support such as ventilation, the ambulance has those equipments. For my wife’s case, it was relatively simple so we just used a taxi. (Case 10, the patient’s husband, the patient suffered from a compound fracture of the tibia).

Because they were so rarely used, many patients and their family did not know about the official ambulance service (115^1^ EMS) while others might have known it but expressed cynicism and disbelief in the service.

No, definitely not [consider calling the 115 Ambulance Service]. You never know [what will happen]. (Case 5, the patient’s friend).

In Vietnam, the main mode of transport to hospital, usually a taxi or motorbike, reflected the patient’s socioeconomic status rather than their injury severity. Generally, patients believed that it was cheaper, faster and more convenient to call a taxi than an ambulance, especially if the site was far away – “*If I cannot even call a taxi, why would an ambulance be any different!”* One of the patients used to work as a driver for the official 115 Ambulance Service, yet he preferred a taxi over an ambulance for the same reasons. Another patient who had used the ambulance service previously concluded that it was better to employ a taxi.

I named the ambulance service “Taxi 115” because they are just transporting service. They are twice or even triple the price of a normal taxi, and with a taxi, I can negotiate. [I noticed that] the ambulance would take at least 15 min while a taxi was there immediately. (Case 26, Male, 50s, metatarsal fracture).

Local newspapers often reported that taxis in Vietnam refused to transport injured patients to the hospital for fear of misfortune, troubles, or blood contamination [14–16] but we found that was not common. One patient reported how a taxi driver had gone above and beyond to help:

First I called my family to inform them of the accident. Then I waved down a taxi. The driver helped bring my motorcycle to a nearby office then personally carried me to the car. He asked me which hospital [I would like to go to], I told him just brought me to the nearest and the best hospital. (Case 33, Male, 60s, tibial fracture).

Some patients reported paying the taxi extra for a “*cleaning fee*”. However, it should be noted that in all the cases when the patients were transported by taxi, they were either conscious or were accompanied by a family member. In one case where the patient was semi-lucid with heavy bleeding, the patient reported that the taxi driver refused to transport him initially but later “*complied”* because of a police presence.

The non-uniform policeman called taxi to bring me to the hospital. At first the taxi driver refused because he could see that there was lots of blood. Then the policeman told him his rank and made the taxi driver do it. (Case 10, Male, 30s, toe fracture).

Apart from transport difficulties other delay in reaching care occurred as patients detoured to nearby health facility for professional opinion and first-aid treatment; as well hoping to avoid going to the hospital. However, these places could offer limited support as this patient demonstrates.

So I went to a pharmacy which was only a few blocks away but they said they cannot do anything. [The bystanders] then told me to go to the community clinic as I felt that the injury was not too bad. When I got there, the nurse had a look [at the injury] and told me to go to the hospital. They did not wrap it up or anything, but at that point, the bleeding has somewhat stopped and the hospital is only 15 min away. (Case 11, male, 70s, compound phalangeal fracture).

### Receiving care

There were hurdles that patients needed to overcome to receive care at a hospital that reflected lack of infrastructure and resources in the health care system. Consequently, finding a suitable hospital could be time-consuming process. For example, in a truck collision, *Long* (M, 70s) injured his head and was left unconscious. A kind bystander promptly brought him to a nearby clinic where he regained consciousness and called his family who took him to Hospital 1. However, the CT Scanner of Hospital 1 was not working and they moved him to Hospital 2 where they took a CT scan. He was then taken to a third hospital for long-term recovery. During this ordeal, the only treatment he had was ‘*a piece of cotton wool in his ears to stop the bleeding*’ (*Long*’s daughter). The overall trip took 2 h and 30 min.

Hospitals did not always provide appropriate treatment. *Huong* (F, 30s, broken arms) fell off her motorbike and was unconscious when her family took her to a small but expensive private hospital. The hospital treated her head wound but missed her broken arm and discharged her. At home, the pain in *Huong’s* arm became so unbearable that she had to go to another hospital. There, *Huong* was put into a queue with other *‘cold/flu patients’.* She *‘lay on the bench holding onto [her] broken arm for half a day’* because the hospital had no protocol to identify patients’ needs.

*Mai* (F, 20s) had similar experience as she was admitted to a nearby hospital promptly following a crash. Her dislocated shoulder was treated but they misdiagnosed the pain in her leg as *‘just swelling and bruising’*. As the pain did not improve she visited another hospital a few days later where she was diagnosed with a torn ligament and recommended surgery costing ‘*80 million dong’* (~ 4000 AUD), an equivalent to 20 months of an average Vietnamese salary, because Mai did not have insurance. *Mai* had the operation 5 months later after she had purchased insurance.

Patients and their families were aware of these problems and take into consideration the hospital reputation, service and location before making any decision. While some would rather travel long distance for a reputable hospital, others opted to go to the nearest place first for initial management and later request a transfer to a more suitable location. The rationale for the latter was that if there is no ambulance to bring a trained technician to help, the patient should go to the nearest place that offered immediate first aid treatment.

The first hospital was about 15 min away from the accident site. The doctors at that hospital were quite good. Although we (the patient and the other party) did not have any money and had to wait for my family to come over to pay, they let me take an X-Ray in the meantime anyway. The X-Ray showed a radial fracture that required surgical fix. I called my aunt who is a nurse at this hospital and she told me to come here instead as it is better and safer here. (Case 9, male, 20s, radial fracture).

Although transferring is common, the process is cumbersome and potential conflicts frequently arise between family and hospital. Unless the patient was formally transferred from a facility that had its own ambulance, they depend on a private vehicle to get to the next hospital. The patient’s family may discharge the patient and take them to another hospital but without a hospital referral letter, the patient might be rejected and be returned to the original hospital. Even when a hospital authorised the transfer, patients might still be required to repeat tests. This process is expensive and takes valuable time needed for treatment.

I want to transfer him [the patient] to a level 1 hospital but this hospital does not authorize the transfer. They told me if I want to move him I can go ahead by my own means […] The hospital has an ambulance but we are not allowed to use it (Case 7’s daughter, patient was 70s Male with head injury).

Throughout these examples (detailed above), the important of family in first response is evident and may be the cause of great delay and inappropriate choice of medical care as family’s decisions may depend on non medical as well as medical variables.

## Discussion

The varied experiences of the forty study patients illuminated substantial complexity relating to social expectations, family function and culturally informed norms and practices. Consequently, the pathway to emergency care for road trauma victims in Vietnam is delayed in each of the principle stages of the three delay model – seeking, reaching and receiving care with potential to negatively impact on patients’ recovery and health.

As noted, patients delayed seeking care as they were frightened of the hospital, misjudged the severity of their injury or were concerned over money and possessions. Previous research has found that treatment provided by Western medical facilities are often perceived as alien, cold and disjointed to many Asian patients [17]. There were also divergences in therapeutic values, expectations and treatment goals among health professionals and their patients, making them wary of seeking care in such facilities [18]. The culture and economic relevance of family was also expressed as patients waited for their family to take the responsibility of transportation to hospital. Although awaiting for the family prolongs time spent at the crash site, this action allows the patient to tap into the family member’s knowledge empowering them to make the most reasonable choice.

Our results revealed many other barriers to reaching care besides lack of transportation to a suitable facility. As in other LMICs, participants were not aware of the official Ambulance Service and did not know that it offered more care than a taxi [19–24]. Participants also expressed distrust over a government-provided service or were concerned about the high cost, slowness to respond, limited coverage, and unclear communication. Evidently, comprehensive education of the public on the operations and activities of ambulance services is needed. It would help to promote the EMS system as well as correct any misunderstanding regarding its use. However, any form of promotional campaign must be accompanied by an actual improvement in services [25]. At the moment, funding poses a substantial barrier to the development of emergency medicine in LMICs including Vietnam [25–27]. It is tempting to emulate high income country model of emergency care, the financial constraints on LMICs will inevitably cause the coverage to be low with little impact on population health. A low income system is more challenging but unavoidable response to the current situation [28].

At the reaching care stage, participants reported they had experienced delay in the quest for first aid treatments at local community facilities such as small medical clinics or pharmacies. Overall they offered little support or expertise for injury care as they lacked resources and skills. Previous first responder trials in Vietnam utilising non health professional community members had demonstrated some success but were limited by ill-defined roles and lack of trust the patients had for volunteer first responders [29]. We noted that staff of the local clinics and pharmacies are embedded in the community, often highly regarded and trusted for their medical knowledge, so it is possible that with suitable support and training, they could act as community-based first responders at the district or village level and coordinate the initial stage of EHS.

Delays in the receiving care stage were also diverse and complex. From the patients’ perspective, we identified three major issues that must be resolved. These are (1) quality of hospital care, (2) transferral process between hospitals and (3) cost and insurance. Participants reported a lack of reliable third stage services and also complained of poor management practices that led to inadequate patient care including no triage and related unnecessary delay. As well participants complained of an opaque (unpredictable) fee schedule and equally confusing insurance policies. Three patients complained of doctors that missed or mis-diagnosed their injuries. Other participants noted many delays and poor quality of care. Indeed, people were willing to wait longer and travel further to a ‘better’ hospital. The stage three problems described here echo previous research in many settings such as Iran [30, 31], Kenya [32] and Zambia [33] where corruption, unfair fees and clinical inexperience discouraged patients from seeking care.

Perceived hospital quality not only reflects hospital conditions and staff skills but also people’s trust in the institution. Previous research has found that less affluent Confucian countries tend to have less general trust as they rely on ‘familism’ as a form of social capital [34, 35]. This was demonstrated in our finding of patients depending on their personal connection at the hospital to judge the hospital quality and trustworthiness. The importance that people place on personalised trust, rather than trust in an institution, explains why one patient found a hospital to be good but another patient reported that it *‘will kill you’*. Therefore, to improve hospital quality, more needs to be done than simply improving technical facilities in a country such as Vietnam. Instead, more attention could be given to improving basic nursing and overall care, building better rapport and enhancing communication with the patients and caregivers.

The between hospital transferral system was found to be important because many patients opted to receive first aid at the nearest hospital then transferred to a more appropriate facility later. Some hospitals have good transfer procedures and ambulances to facilitate such process but others do not. Regardless of patient injuries and hospital levels, some patients could easily get transferred while others were refused which resulted in unnecessary tension between the hospital and family. A more streamlined procedure and better communication strategy to inform the patient of the risks and benefits of transferring might help relieve the tension and improve patient’s experience.

### The three-delay model

For our analysis of patient pathways to care, the three delay model enabled us to organise the information and make recommendations for future development. However, the three delay model does not entirely capture the complexity of the sociocultural experience of patients and their families. Our study found that attaining prehospital care for traffic injured patients was a round-about where patients circulate between the crash site, several hospitals and home. Furthermore, the three delay model focuses superficially on issues with accessing care while overlooking the sociocultural environments that inform people’s care-seeking processes for traffic injured patients in Vietnam. Therefore, any recommendations to improve the emergency care need to take into consideration these sociocultural contexts.

### Study strengths and limitations

Our research design was qualitative and this allowed us to explore in-depth the thought processes and complex sociocultural structures that shaped the data that we gathered. We recruited participants in orthopaedics and neurosurgical wards as these are the places where most traffic injured patients hospitalised but such a recruitment method did attract more fracture and restricted our insights on other forms of injury. Available time and resources limited the fieldwork to two urban locations. Urban dwelling Vietnamese are likely to be relatively less poor and have greater access to care resources. The study sites are two historically and culturally diverse areas and the participants came from different socioeconomic backgrounds. Such diversity enabled capture of different views, beliefs and practices that added to the study’s depth and generalisability. Furthermore, our in-depth interviews on pathway to care were conducted by a cultural insider (TT). This made it possible to gain a fine-grained view of the participants’ experiences and allowed us to examine the influences of sociocultural structures on the patient care seeking processes. We found the widespread importance of family to the injury outcome as well as the primary healthcare.

## Conclusions

Our data showed that sociocultural characteristics profoundly influence traffic injured patient pathways to emergency care in Vietnam. As in other developing countries without an efficient EMS system, Vietnamese patients rely on family, bystander and social networks to receive the care they need. For traffic crashes we studied, the interactions among bystander, patients and their families and hospital personnel were complex. Indeed, sociocultural backgrounds in Vietnam delay and impede EMS care. Any future EMS developments in the country need to be aware of these elements and design accordingly to provide effective and culturally appropriate care for traffic injuries in Vietnam.

## Supplementary Information

**Additional file 1: Supplementary 1**. Consolidated criteria for reporting qualitative studies (COREQ): 32-item checklist.

**Additional file 2: Supplementary 2**. Interview guide (translated from Vietnamese).

## Data Availability

The data that support the findings of this study are available on request from the corresponding author [TT] for collaborative work. The data are not publicly available due to them containing information that could compromise research participant privacy/consent.

## Abbreviations

EMS: Emergency medical service
EHS: Emergency health service
HCMC: Ho Chi Minh City
HIC: High income country
LMIC: Lower-middle income country

## Footnotes

1 One hundred fifteen is the official phone number given to the medical emergency service in Vietnam and is widely used as the name of the service
