# Supplementary material for: Pathways to care: a case study of traffic injury in Vietnam
Source: BMC Public Health. 2021 Mar 16;21:515. doi: 10.1186/s12889-021-10539-9 (PMC7968285; doi:10.1186/s12889-021-10539-9)
Supplement: Supplementary file 1 — Additional file 1: Supplementary 1. Consolidated criteria for reporting qualitative studies (COREQ): 32-item checklist. [file 12889_2021_10539_MOESM1_ESM.docx]

# Supplementary 1 – Consolidated criteria for reporting qualitative studies (COREQ): 32-item checklist

Adapted from: Tong A, Sainsbury P, Craig J. Consolidated criteria for reporting qualitative research (COREQ): a 32-item checklist for interviews and focus groups. International journal for quality in health care. 2007;19(6):349-57.

| **No. item** | **Guide questions/Description** | **Response** |
| --- | --- | --- |
| Domain 1: Research team and reflexivity | | |
| Personal Characteristics | | |
| 1. Interviewer | Which author/s conducted the interview or focus group? | TT conducted all the interviews in Vietnamese.  (see Methods p. 5-6) |
| 2. Credentials | What were the researcher’s credentials? E.g. PhD, MD | TT: BSc, MPH, MD  AS: MBBS, MPH, MD, DTM&H, FRCP  CB: DipT, BA, MA, PhD |
| 3. Occupation | What was their occupation at the time of the study? | TT: PhD scholar and medical graduate  AS: Professor of epidemiology and public health  CB: Professor in medical anthropology |
| 4. Gender | Was the researcher male or female? | Female: TT, CB. Male: AS |
| 5. Experience and training | What experience or training did the researcher have? | All researchers had experience with qualitative and quantitative research methods based on previous research projects and training. |
| Relationship with participants | | |
| 6. Relationship established | Was a relationship established prior to study commencement? | No. All participants were recruited during fieldwork and interviews followed soon afterward.  (More information can be found in Methods section, page 5-6) |
| 7. Participant knowledge of the interviewer | What did the participants know about the researcher? e.g. personal goals, reasons for doing the research | The participants knew that the interviewer was a PhD scholar from Australia and the research is part of her PhD. |
| 8. Interviewer characteristics | What characteristics were reported about the inter viewer/facilitator? e.g. Bias, assumptions, reasons and interests in the research topic | The interviewer has interests in injury research with some medical knowledge. At the time of the interview, TT has not graduate from medical school. |
| Domain 2: study design | | |
| Theoretical framework | | |
| 9. Methodological orientation and Theory | What methodological orientation was stated to underpin the study? e.g. grounded theory, discourse analysis, ethnography, phenomenology, content analysis | Focus ethnography with in-depth interviews and direction observation. Three delay model was used as a framework for data analysis.  (See Methods page 6) |
| Participant selection | | |
| 10. Sampling | How were participants selected? e.g. purposive, convenience, consecutive, snowball | Opportunistic sampling was used. Suitable participants were introduced by their treating doctor or nurses.  (See Methods page 5) |
| 11. Method of approach | How were participants approached? e.g. face-to-face, telephone, mail, email | The participants were approached face-to-face.  (See Methods page 6) |
| 12. Sample size | How many participants were in the study? | 48 interviews were carried out. 40 with patients and 8 with families.  (See Table 1 for participants characteristic) |
| 13. Non-participation | How many people refused to participate or dropped out? Reasons? | Five people refused to participate. No reason was given. |
| Setting | | |
| 14. Setting of data collection | Where was the data collected? e.g. home, clinic, workplace | The interviews took place by patient’s bed or at quiet corners in the hospital. |
| 15. Presence of non-participants | Was anyone else present besides the participants and researchers? | Participant’s family might be present at time of interview. |
| 16. Description of sample | What are the important characteristics of the sample? e.g. demographic data, date | Characteristics of participants were outline in Methods section table 1 |
| Data collection | | |
| 17. Interview guide | Were questions, prompts, guides provided by the authors? Was it pilot tested? | The interview guide is enclosed with the manuscript in Supplementary. |
| 18. Repeat interviews | Were repeat interviews carried out? If yes, how many? | Some participants were interviewed a few times over a couple of days. This was due to time constrained as the participants had to leave for treatments or other obligations. This also give the interviewers time to build up trust and establish better rapport with the participants and their families. |
| 19. Audio/visual recording | Did the research use audio or visual recording to collect the data? | All interviews were audio recorded with permission from the participants .  (Page 6) |
| 20. Field notes | Were field notes made during and/or after the inter view or focus group? | Short field notes were made during and after the interviews.  (Page 6) |
| 21. Duration | What was the duration of the inter views or focus group? | The interviews last approximately one-hour.  (Page 6) |
| 22. Data saturation | Was data saturation discussed? | Data saturation was discussed among the research team and considered sufficient to perform the analysis. |
| 23. Transcripts returned | Were transcripts returned to participants for comment and/or correction? | Transcripts were not returned to the participants. |
| Domain 3: analysis and findings | | |
| Data analysis | | |
| 24. Number of data coders | How many data coders coded the data? | TT coded the data  (Page 6) |
| 25. Description of the coding tree | Did authors provide a description of the coding tree? | Coding tree was summarized as Table 2. |
| 26. Derivation of themes | Were themes identified in advance or derived from the data? | Themes were derived from the data but guided by literature and arranged according to the three delay models.  (See Methods page 6) |
| 27. Software | What software, if applicable, was used to manage the data? | ATLAS ti 8 was used to manage the data.  (Page 6) |
| 28. Participant checking | Did participants provide feedback on the findings? | It was not possible to engage participants to provide feedback regarding the research findings. To protect participants’ identify, the researcher did not seek any personal information other than name and age group. The participants however were provided with an information sheet containing TT’s contact details and were encouraged to contact TT shall they require further information. |
| Reporting | | |
| 29. Quotations presented | Were participant quotations presented to illustrate the themes/findings? Was each quotation identified? e.g. participant number | Yes. Participants were given pseudonyms or numbered identifier, their gender and age groups were also provided. |
| 30. Data and findings consistent | Was there consistency between the data presented and the findings? | Yes. |
| 31. Clarity of major themes | Were major themes clearly presented in the findings? | Yes. |
| 32. Clarity of minor themes | Is there a description of diverse cases or discussion of minor themes? | Few diverse cases and minor themes are described in the results section. |
