# Supplementary material for: Pathways to care: a case study of traffic injury in Vietnam
Source: BMC Public Health. 2021 Mar 16;21:515. doi: 10.1186/s12889-021-10539-9 (PMC7968285; doi:10.1186/s12889-021-10539-9)
Supplement: Supplementary file 2 — Additional file 2: Supplementary 2. Interview guide (translated from Vietnamese). [file 12889_2021_10539_MOESM2_ESM.docx]

**Supplementary 2 – Interview guide (translated from Vietnamese)**

The purpose of this discussion is to discuss about:

- Your thoughts and feelings about traffic accidents and helping others.
- Your crash experience and your experiences with the emergency medical service system and care.

**Your feelings, thought and belief**

- What kind of vulnerabilities do you think injured patients are exposed to at the scene of injury?
- What are the major worries related to injured patient (As relative? As layperson?)
- What are the main concerns relating to calling ambulance?
  - Prompt: Have you call before? What happened?
- What are the major concerns relating to sending the patient to the hospital by yourself (As relative? As layperson)
- What are the major worries related to helping patient?
- What kind of vulnerabilities do you think helpers are exposed to (at the scene of injury? afterward?)
- How do you decide whether to help or not to help?
  - Prompt: What are the main factors determine the process of deciding to help or not to help (relating to the patient, the environment, and the helper)?

**Your experiences**

- Can you tell me a little bit about what happened?
- What were you doing when the crash occurred?
- Who was involved in response to the incident? The first person respond the only one in the scene? Did other people see and did not stop to help??
- What was done?
- *How long did it take to the hospital? (patients)/ How long did it take before they were taken to hospital? (witnesses)*
- How did you/patient get to the hospital? Who decides
- Why particular mode of transport was employed?
- What happened at the hospital?
- What could have been done differently?
